# Factors Associated with Injuries among Commercial Motorcyclists: Evidence from a Matched Case Control Study in Kampala City, Uganda

**DOI:** 10.1371/journal.pone.0148511

**Published:** 2016-02-26

**Authors:** Nazarius M. Tumwesigye, Lynn M. Atuyambe, Olive K. Kobusingye

**Affiliations:** 1 Department of Epidemiology and Biostatistics, Makerere University School of Public Health, Kampala, Uganda; 2 Department of Community Health and Behavioral Sciences, Makerere University School of Public Health, Kampala, Uganda; 3 Department of Disease Control and Environmental Health, Makerere University School of Public Health, Kampala, Uganda; Beihang University, CHINA

## Abstract

**Introduction:**

Road traffic injuries are the eighth leading cause of death globally and the most affected are young people aged 15–29. By 2030 road traffic deaths will become the fifth leading cause of death unless urgent action is taken. Motorcyclists are among the most vulnerable road users and in Uganda they contribute 41% of all road traffic injuries. This paper establishes factors associated with the injuries of commercial motorcycle riders also known as boda-boda riders in Kampala, Uganda’s capital city.

**Methods:**

The study was matched case-control with a case being a boda-boda rider that was seen at one of the 5 major city hospitals with a road traffic injury while a control was a boda-boda rider that was at the parking stage where the case operated from before the injury. The sample size was 289 riders per arm and data collection took 7 months. A structured questionnaire was used to collect data on background and exposing factors. Being matched case-control data conditional logistic regression was used in the analysis.

**Results:**

Factors independently associated with injury among motorcyclists were younger age group, being a current alcohol drinker (OR = 2.30, 95%CI: 1.19–4.45), lower engine capacity (<100cc)(OR = 5.03, 95%CI: 2.91–8.70), riding experience of less than 3 years, not changing a motorcycle in past 1 year (OR = 2.04, 95%CI: 1.19–3.52), riding for a longer time in a day (OR = 6.05, 95%CI: 2.58–14.18) and sharing a motorcycle (OR = 8.25, 95%CI:2.62–25.9). Other factors associated with injury were low level of knowledge of traffic rules, being stopped by police for checks on condition of motorcycle/license/insurance, working till late.

**Recommendations:**

More road safety sensitization is required among riders to raise awareness against sharing motorcycles, working for a longer time and alcohol consumption. Police enforcement of drink-driving laws should include riders of commercial motorcycles. Investigate the validity of motorcycle riding licenses and test the riding competency of all who got licenses in last 3 years.

## Introduction

Road traffic injuries are the eighth leading cause of death globally and the leading cause of death among young people aged 15–29 [1]. Nearly 3,400 people die on the world's roads every day[1]. A disproportionate burden is borne by low- and middle-income countries (LMICs) and vulnerable road users such as pedestrians, cyclists, and riders of motorcycles [2]. Current trends suggest that by 2030 road traffic deaths will become the fifth leading cause of death unless urgent action is taken[3]. Road traffic injuries are estimated to cost LMICs between 1–2% of their gross national product, estimated at over US$ 100 billion a year[4]. The rate of road traffic injuries in LMICs is twice as much as that in high-income countries. The African region has the highest road traffic fatality rate (24.1 per 100,000 population)[1]. Motorcyclists are among the most vulnerable road users. Nearly a quarter (23%) of the world’s road traffic deaths occurs among motorcyclists. Per vehicle mile traveled, motorcycle riders have a 34-fold higher risk of death in a crash than people driving other types of motor vehicles[5].

The commonest injuries among motorcyclists are lower-extremity and head injuries[5]. Other accident related effects may range from minor abrasions, to fractures and spinal deformations [6–7]. According to the USA federal government, per mile traveled in 2006, there were 35 times more deaths from motorcycle accidents than from car accidents[8]. Motorcycles face higher dangers from several different road hazards than do cars and other vehicles[9]. Due to the smaller size and less stable nature of the motorcycle, potholes, dead animals, slick pavement conditions, uneven heights between lanes, and other irregularities or unexpected objects in the road pose a serious safety threat to motorcyclists. Again, because the motorcyclist is not surrounded by a metal case and is likely to be thrown far and hard, such crashes are more deadly than those involving other vehicle types. Motorcycle injuries constitute a major but neglected public health problem in rapidly motorizing LMICs, and the relevant risk factors have not been adequately examined in these countries[5].

Following the publication of the 2009 global status report on road safety *[10]* the United Nations General Assembly (UNGA) adopted a resolution [11] that declared 2011–2020 the decade of action for road safety. Among other things the report showed that the Africa region had one of the highest rates of road traffic mortality. This study will contribute evidence that will help in the fight against one of the most vulnerable road user categories targeted for action during this decade of action for road safety.

Studies carried out in several countries have established that factors that have an influence on motorcycle crashes include age, ethnicity, income, education, motorcycle license insurance status, self reported alcohol consumption in the 12 hours preceding the crash, years of on-road riding experience, kilometers travelled on the specific motorcycle at interview, posted speed limit, and weather conditions[12–17]. Other factors are engine size, time of day[18], ownership, speed and risk taking behavior[5]. An extensive search of journal articles on motorcycle injuries in Uganda showed lack of data on factors associated with boda-boda injuries. Most studies carried out in the country have produced a descriptive analysis of data from victims of the boda-boda crushes with little or no effort to identify predisposing factors[19–20].

In Uganda, motorcycle taxis *(boda-boda)* are a major cause or agent of road traffic injuries and a significant economic burden as each costs an estimated range of USD 300–369 in treatment [19–20]. A study of July to September 2001 records in emergency wards of Mulago National referral hospital found boda-boda crashes contributed 25% of the road traffic injuries(RTI) while another records based study in 2008 found they contributed 41% of the RTIs [20]. Boda-bodas contribute 21.4% of all physical trauma cases in Gulu regional referral hospital[21]. The costs attributable to boda-boda injuries in Mulago national referral hospital make up approximately 4.2% of the total hospital budget, 15% of the hospital health services budget and 62.5% of the hospital’s budget allocation for directorate of surgery[20]. The percentage of accidents attributable to Boda-bodas has been increasing annually [20]. They are a leading cause of accident scene fatalities in Kampala[22]. About 17% of 2954 people killed in RTI in 2010 were operators or passengers of two or three wheeled vehicles, primarily motorcycles[23].

Boda-bodas are filling a gap in the transport system in Uganda. They operate where more conventional transport services are uneconomical or physically impossible [24]. Therefore, the boda-boda mode of transport is likely to remain one of the most important players in the transport sector for quite a while and this calls for increased efforts to make it safer. This paper adds on existing evidence that can aide intervention to make the boda-boda transport safer.

The increase in number of *boda bodas* has raised road safety concerns as the number of injuries attributed to them continues to rise. This study aims at establishing the factors associated with motorcycle injuries in Kampala city. In the process it also establishes whether factors associated with motorcycle injuries in studies elsewhere are mirrored in Uganda.

## Materials and Methods

### Study setting

The study was carried out at Mulago national referral hospital and four other major city hospitals, namely, Kibuli, Nsambya, Rubaga and Mengo. Mulago is a public facility offering free services while the rest are private hospitals where patients have to pay for services. Owing to free services offered at Mulago the hospital is overstretched with large number of patients for limited facilities such as in-patient beds[25]. Data collection took place between March and September 2014. In each of the hospitals the research team worked closely with the emergency department. Controls were sourced from the roadside boda boda stages. A stage is a recognized location where boda boda riders wait for their clients. They are highly organized, with management structures to ensure that only those riders registered to operate at a designated stage do work from there.

### Design

The research was designed as matched case-control study with cases being all those boda-boda riders who were injured on the road and brought to the study hospitals. The controls were boda-boda riders at the same boda-boda packing stage as the cases. Therefore, the matching was by packing stage. The ratio of cases to controls was 1:1. It was assumed that both controls and cases were exposed to similar conditions that could influence their risk of injury.

### Study population

The population under study comprised boda-boda riders in Kampala city. They were grouped into two: the cases that were injured at the time of the study and controls that were at parking stages where the cases operated from. Since the hospitals are the largest in Kampala city, comprising of both public and private facilities, it is believed that the cases and the selected controls represent all boda-boda riders in the city.

### Sample size and selection of participants

The sample size was 289 motorcyclists per arm. Using an OPEN EPI software which applies the formula by Fleiss[26], aiming to detect a difference of 12% (absolute between exposure in controls and cases), a power of 80%, a ratio of cases and controls of 1:1 then the minimum sample size would be 259 per arm. An exposure variable used in the computation was alcohol consumption whose prevalence is 55%[27] among men in general population. When a 10% non-response rate was factored in the sample size the final figure became 289 per arm.

The method of selecting a control was informed by the pilot test which found that sampling the eligible boda-boda riders on a stage was ineffective. Boda-boda riders were found to be impatient at the sight of a potential client, and some would abandon the interview midway if a client arrived. Some control riders were not willing to participate in the study because they were unsure of the outcome of the study despite several re-assurances. Shortly before the study Kampala Capital City Authority had registered all boda-boda riders with the aim of streamlining their operations. Some thought the data collection was linked to the registration process which would eventually limit their operations. For these reasons, it was decided that the first of the eligible controls to read through the consent form and consent for the interview would be interviewed.

### Inclusion and exclusion criteria

Controls had to be known usual boda-boda operators at the stage while the cases had to have been brought to hospital after a boda-boda related injury. Cases would have been stabilized after initial emergency treatment. Both cases and controls must have been operating within the boundaries of Kampala city. The exclusion criteria were refusal to participate or complete the interview and deteriorating condition of the cases.

### Data collection process

The main place of data collection was the emergency department and therefore the nurse in-charge of the department was instrumental in ascertaining the cases. The data collection was by face-to-face interviews using a structured questionnaire. Each control was interviewed within 24 hours after interview of the case. The interviewers were knowledgeable in the local language. At the boda-boda packing stage the first contact was the stage leader. At each stage the data collector worked with the stage leader to identify eligible riders.

### Variables and source of data

The main outcome variable was a boda-boda related injury. The key exposure variables were taking alcohol, frequency of alcohol consumption, Alcohol Use Disorders Identification Test (AUDIT) score and helmet use. Major confounding variables included background characteristics (age, marital status, education, religions and income) engine capacity, ownership of motorcycle and experience of riding, helmet and reflective jacket use, training, time of start and end of riding in a day, model of the motorcycle and ownership of the motorcycle, alcohol and drug use and knowledge of road safety rules. Inclusion of each of the variables in the study is premised on results of previous studies carried out in different parts of the world as shown in the background section.

Data on most variables were obtained from face-to-face interviews. Data on boda-boda related injury were obtained through observation, enquiries from the nurse in-charge at the emergency department and interview of the rider. The data on exposure and confounding variables were obtained through face-to-face interviews with the boda-boda riders. The data on AUDIT were obtained using a tool developed with support of World Health Organization[28]. The tool was embedded within the questionnaire.

### Potential bias and efforts to address them

Selection of controls had potential for bias towards the educated riders and those who may not have had many clients. This is because those who accepted to read and sign the consent forms were more likely to have been educated. In addition, those who accepted to be interviewed may have been those who were either not busy or hoped to get money for compensation of their time. To counteract this bias, research assistants were trained to explain the study well and to use local language. The tool had local translation as well.

A rider who has not had an injury at around the same time his colleague is in hospital may not be the best control because he may have had an injury days or weeks before or after. However, these control riders at the stages provide the best base for comparisons and they are also easy to get.

Thirdly, those who go to hospitals may not be the best representation of those who are involved in road crashes and eventual injury. For example, there are those will only go to hospitals when they are severely injured. Then the fact that most of the injured motorcycle riders were recruited from the public hospital is another cause for selection bias. The hospital is the only one that offers free services. It is possible that boda-boda riders, who are generally low income earners, were either taken to, or preferred to go where they could access free services. The effort to collect data from other major but paying hospitals was meant partly to address the bias created by use of one source of data.

Recall bias and social desirability bias from interviewer-administered surveys are also important potential limitations. For example riders may hesitate to report they take alcohol given that it impairs decision making during riding.

Residual confounding may arise from misclassification of measured covariates as well as unmeasured variables. Examples of unmeasured variables are distance covered, fatigue, road features and environmental factors.

### Data management

All data were double entered using EPIDATA V3 software and later exported to STATA V12 for cleaning and analysis. The data entry screens had been fitted with range and consistency checks.

Data from a few of the variables needed further treatment before use in analysis. Key among these were the AUDIT score and background characteristics. The audit score is a summative one and it involved adding up scores for all the ten questions. The score was categorized into low (0–7), medium (8–15) and high (16–40) as informed by documents on AUDIT score[28]. Other categorization was made with the aim of having sufficient number of respondents for comparison between categories. Examples were 5 year age groups for age and two categories of marital status (married versus not married).

### Data analysis

To establish factors associated with injury, bivariate and multivariable conditional logistic regression analyses were carried out with the outcome as case/control. Conditional logistic regression was used because the cases and controls were matched. Model building principles [29] were followed in getting the final statistical model. These included parsimony and maximizing goodness of fit. Wald’s tests guided the backward elimination method that was used in model building. The criteria for selecting variables into the multivariable model were p-value of 0.1 and less while exclusion criteria was p = 0.05. The goodness of fit of the model was monitored by increase in the value of the log likelihood and p-values of the coefficients because available common standard statistical packages do not have diagnostic statistics for matched designs [30]. Item non-response was nil for most of the key variables and very minimal (<2%) where it existed. Given that non-response was very minimal and we had adjusted for it in the design of the study we did not make any further adjustment in analysis.

Specifically, the analysis was carried out in STATA V12 using the *clogit* command to fit the fit the model:

$$\text{logit}(p)=\alpha_{1}+\alpha_{2}x_{2}+\ldots \ldots .+\alpha_{m}x_{m}+\beta_{1}x_{1}+\ldots .+\beta_{k}x_{k}$$ (1)

Where

m = number of confounder adjustment variables

*α* + *α*_2_*x*_*s*2_ +…….+ *α*_*m*_*x*_*m*_ = variables for many small strata which are not of interest per se[31]. Strata are made of pairs of cases and controls.

$$\beta_{1}x_{1}+\ldots .+\beta_{k}x_{k}=\;\text{Variables of interest and any additional adjustment variables}$$

For each exposure variables $x_{1},\ldots \ldots .x_{k}\;x_{E}=\{{}_{0Not\;\text{exp}\;osed}^{1Exposed}$ for matched pairs

$$x_{2}=\left\{ \begin{array}{l} 1\;\text{pair}\;2\\ 0\;\text{Else} \end{array}\right.,\;x_{3}=\left\{ \begin{array}{l} 1\;\text{pair}\;3\\ 0\;\text{Else} \end{array}\right.,\ldots \ldots \ldots \ldots \ldots \ldots \ldots \ldots ,x_{m}=\left\{ \begin{array}{l} 1\;\text{pair m}\\ 0\;\text{Else} \end{array}\right.$$

Using the model building methods outlined above three statistical models were developed. The first model examined the relationship between boda-boda related injury and background characteristics, the second was the grand model that highlighted all factors associated with the injury while the third model only showed how the AUDIT score, index for knowledge of recommended riding practices and enforcement of traffic rules are related to boda-boda related injury. None of these factors featured in the second model because their relationship with boda-boda injury was grossly confounded by other variables yet they represent whole sections of the tool used.

The minimal data set underlying the main multivariable findings in the study is accessible through a link to a STATA file named S1 Dataset.

### Ethical considerations

The investigators first secured ethical approval from Makerere University School of Public Health Institutional Research Review Board (IRB) and the National Council of Science and technology (NCST).

Written consent was requested from each eligible rider after a clear explanation of the research project, its goal, and objectives and eventual possible benefits to road users. The data collectors assured the study participants of the confidentiality of the interviews and the security of the data, which could only be accessed by the researchers on this study. All the riders interviewed provided written informed consent.

## Results

### Study participants

**Fig 1:**
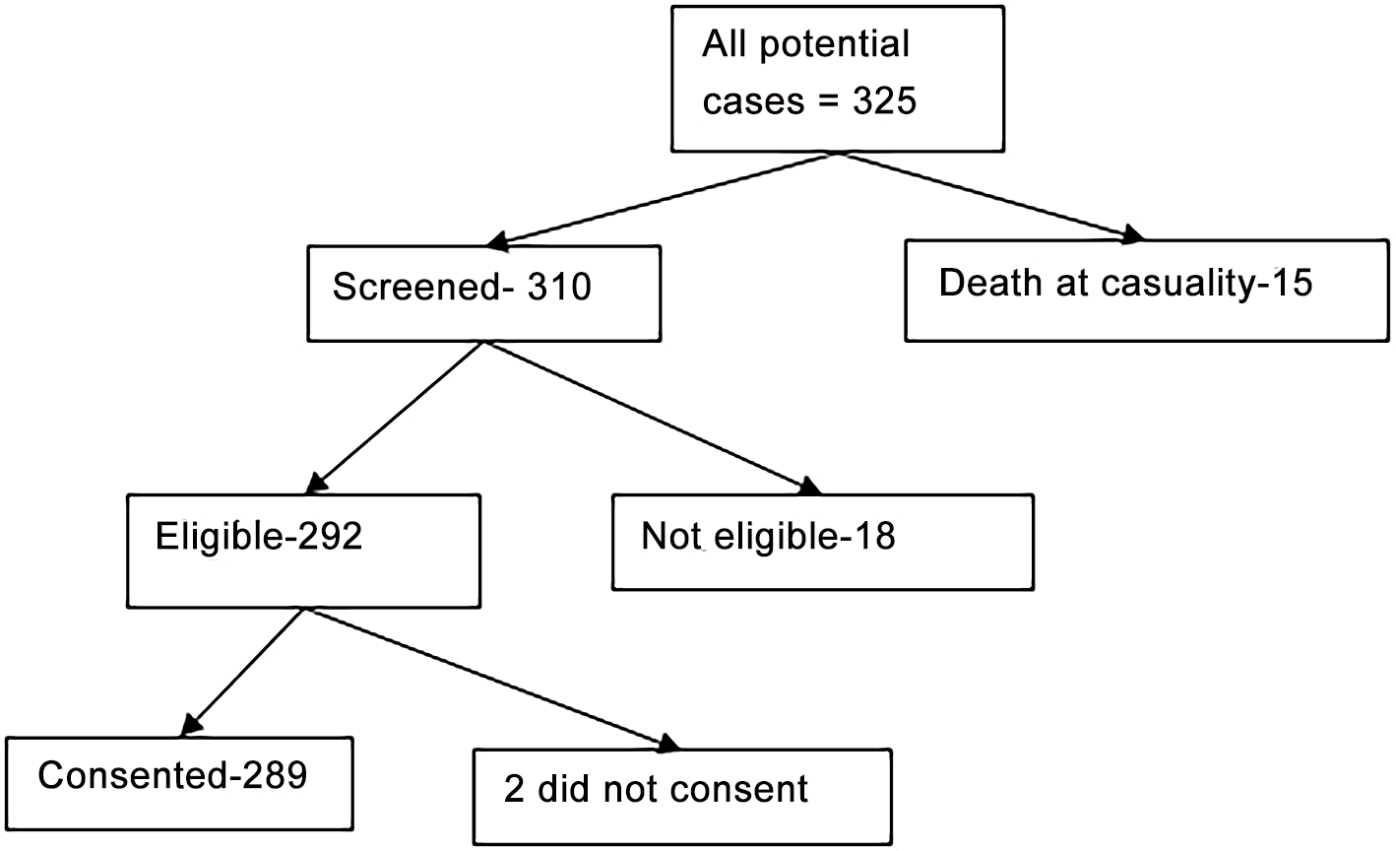
The first table shows the background characteristics of the cases and controls and tests the significance of the difference between the two groups. The second table focuses on risky practices and related un-adjusted odds ratios for getting a motor-cycle injury. The rest of the tables have results from multivariable analysis.

The total number of cases that were potentially eligible for the study was 325 and it included 15 who died at emergency department shortly after arrival (Fig 1). Those screened for eligibility were 310 and included 18 who were ineligible because information on motorcycle relatedness of the injury could not be ascertained. Of the 292 that were eligible 2 did not consent. Those confirmed eligible were 289.

### Background characteristics of cases and controls

The study participants were equally distributed across the five divisions of Kampala city. The age ranged from 18 to 56 years (Table 1). Most of respondents were in the age group 25–34 (cases: 56%, controls: 57%), were married (cases: 66%, Controls: 81%), had attained at least primary education (cases: 80%, controls: 84%), were Christian (cases: 81%, controls: 70%) and earned less than Shs 80,000 (USD 30) per week (cases: 64%, controls: 80%).

**Table 1:** Background characteristics of the cases and controls.

Characteristics	Cases n(% of 289)	Controlsn(% of 289)	OR (95%CI)^a^
**Division**			
Central	84(29.1)	84(29.1)	**--**
Kawempe	62(21.5)	62(21.5)	**--**
Makindye	55(19.0)	55(19.0)	**--**
Nakawa	48(16.6)	48(16.6)	**--**
Rubaga	40(13.8)	40(13.8)	**--**
**Age**			
18–24	79(27.3)	39(13.5)	1.0
25–29	86(29.8)	99(34.3)	0.39(0.23–0.65) ***
30–34	77(26.6)	64(22.2)	0.58(0.34–0.99)*
34–39	29(10.0)	48(16.6)	0.28(0.15–0.53)***
40+	18(6.2)	39(13.5)	0.22(0.11–0.45)***
**Marital status**			
Single/separate/widow	98(33.9)	54(18.7)	1.0
Married	191(66.1)	235(81.3)	0.43(0.29–0.64)**
**Education**			
None	59(20.4)	46(15.9)	1.0
Primary	114(39.5)	118(40.8)	0.73(0.45–1.19)
Secondary-S1-S4	98(33.9)	102(35.3)	0.72(0.44–1.19)
A level& tertiary	18(6.2)	23(8.0)	0.60(0.29–1.24)
**Religion**			
Catholic	122(42.7)	95(32.9)	1.0
Protestant	80(28.0)	73(25.3)	0.82 (0.54–1.25)
Muslim	54(18.9)	87(30.1)	0.47(0.30–0.74)**
Other Christian	30(10.5)	34(11.8)	0.69(0.40–1.18)
**Income per week in Shs(USD)**			
<30,000 (<13)	17(5.9)	71(24.7)	1.0
30,000–49,999(13–20)	48(16.6)	80(27.9)	2.42(1.22–4.83)*
50,000–79,999(20–30)	119(41.2)	79(27.5)	5.97(3.12–11.4)***
80,000–99,999(30–40)	13(4.5)	17(5.9)	3.34(1.27–8.78)*
100,000+(40+)	92(31.8)	40(13.9)	12.93(5.95–28.08)***

-- Not applicable since no variation

^a^Obtained from conditional logistic regression

*p<0.05

**p<0.01

***p<0.001

Examination of crude odds ratios of getting injured in different categories compared to base categories shows that the odds of getting injured significantly reduced with higher age groups (OR = 0.22, 95%CI:0.11–0.45), were lower among the married (OR = 0.43, 95%CI:0.29–0.64), lower among the Muslims (OR = 0.47, 95%CI: 0.30–0.74) and highest among high income earners (>Shs 100,000 per week, equivalent to US$40) (OR = 12.9, 95%CI: 5.95–28.08). The odds of injury did not significantly change by education level.

### Modifiable Risky factors associated with motorcycle injury

Table 2 shows results of bivariable analysis. The major modifiable risk factors associated with motorcycle injuries were found to be being current drinker (OR = 1.97, 95%CI: 1.31–2.96), lack of ownership of the motorcycle (OR = 2.79, 95%CI: 1.90–4.09), lower engine capacity (OR = 5.11, 95%CI:3.41–7.66), short time of riding experience (<2 years)(OR = 4.18, 95%CI: 2.64–6.58), failure to change a motorcycle in a year (OR = 1.96, 95%CI: 1.37–2.80), lack of training on riding (OR = 4.04, 95%CI: 2.65–6.15), starting riding before 6am (OR = 3.02, 95%CI:1.87–4.86), riding till late (OR = 3.88, 95%CI: 2.25–6.69) sharing motorcycle (OR = 4.88, 95%CI: 2.28–10.43), inconsistent/non-use of helmet (OR = 2.21, 95%CI: 1.47–3.30), low knowledge of road safety rules (OR = 11.4, 95%CI: 5.8–22.5), having ever been stopped by traffic police for checks on condition of motorcycle/ license/insurance (OR = 3.56, 95%CI: 2.50–5.08). Factors that did not have any relation with road traffic injury among the riders in bivariate analysis include speed, type of motorcycle, having a motorcycle driving permit.

**Table 2:** Road safety practices and injury experience- Bivariable analysis.

Practices	Casesn (%)	Control n (%)	UOR^a^
**Alcohol consumption**			
No	203(70.2)	237(82.0)	1
Yes	86(29.8)	52(18.0)	1.97(1.31–2.96)**
**How often did you take alcohol in last 12 months**			
Never	210(72.7)	236(81.7)	**1**
Monthly or less	29(10.0)	9(3.1)	3.56(1.65–7.66)**
2–4 times a month	39(13.5)	20(6.9)	2.43(1.32–4.50)**
2+ times a week	11(3.8)	24(8.3)	0.45(0.17–1.18)
**Ownership of motorcycle**			
Yes	99(34.3)	162(56.1)	1
Yes, co-own it	27(9.3)	28(9.7)	1.52(0.84–2.74)
No	163(56.4)	99(34.3)	2.79(1.90–4.09)***
**Type of motorcycle**			
Bajaj	263(91.0)	274(94.8)	1
Other	26(9.0)	15(5.2)	1.92 (0.95–3.85)
**Engine Capacity**			
100+	122(42.2)	237(82.0)	1
50–99	167(57.8)	52(18.0)	5.11 (3.41–7.66)***
**Experience of riding**			
More than 2 years	179(61.9)	252(87.2)	1
0–2 years	110(38.1)	37(12.8)	4.18(2.64–6.58)***
**Number of years of riding**			
0–2	110(36.1)	37(12.8)	1.0
3–4	74(25.6)	62(21.5)	0.44 (0.26–0.76)**
5–7	73(25.3)	82(28.4)	0.31(0.18–0.54)***
8+	32(11.1)	108(37.4)	0.10(0.05–0.19)***
**Changed motorcycle in past 1 year**			
Yes	83(28.7)	126(43.6)	1
No	206(71.3)	163(56.4)	1.96(1.37–2.80)***
**Received rider training**			
Yes	172(59.5)	254(87.9)	1
No	117(40.5)	35(12.1)	4.04(2.65–6.15)***
**Time of starting of riding**			
8+	69(25.8)	110(38.3)	
6–7	96(36.0)	125(43.6)	1.29(0.83–2.00)
Before 6am	102(38.2)	52(18.1)	3.02 (1.87–4.86)***
**Time of end of riding**			
1:00–7:59pm	65(22.5)	101(35.0)	1
8:00–8:59pm	56(19.4)	88(30.5)	0.96(0.57–1.60)
9:00–9:59 pm	46(15.9)	62(21.5)	1.09 (0.67–1.77)
10-12pm	89(30.8)	32(11.1)	3.88(2.25–6.69)***
Morning next day	33(11.4)	6(2.1)	8.03(2.98–21.67)***
**Length of time of riding (hours)**			
<12	54(18.7)	68(23.5)	1
12	43(14.9)	67(23.2)	0.89(0.52–1.53)
13	51(17.7)	75(26.0)	0.85(0.51–1.42)
14–19	141(48.8)	79(27.3)	2.29(1.43–3.68)**
**Shares motorcycle**			
No	246(85.1)	276(95.8)	1
Yes	43(14.9)	12(4.2)	4.88(2.28–10.43)***
**Helmet use**			
Always	196(67.8)	237(82.0)	1
Not always/doesn’t use	93(32.2)	52(8.0)	2.21(1.47–3.30)***
**Alcohol problem Audit score**			
Low (0–7)	247 (85.5)	271(93.8)	1
Medium (8–15)	27(9.3)	14(4.8)	1.93(1.01–3.68)*
High (16+)	15(5.2)	4(1.4)	3.75(1.24–11.3)*
**Knowledge of road safety rules scores**			
High (21–27)	19(6.64)	99(34.5)	1
Medium (19–20)	39(13.6)	71(24.7)	3.62 (1.71–7.66)**
Low (10–18)	228(79.7)	117(40.8)	11.4 (5.8(22.5–54)***
**Ever been stopped by a policeman to check mechanical condition?**			
No	81(28.0)	181(62.6)	1
Yes	208(72.0)	108(37.4)	3.56 (2.50–5.08)***

^a^ Obtained after conditional logistic regression

* p<0.05

**p<0.01

*** p<0.001

### Multivariable analysis

In a model with only background (socio-demographic) characteristics, it was found that factors associated with injury were lower age group, not being married and having a higher income (Table 3). The background variables in the study are age, marital status, education level, religion and income.

**Table 3:** Background characteristics and injury experience- multivariate analysis.

Characteristics	Cases n(% of 289)	Controlsn(% of 289)	OR (95%CI)^a^	AOR (95%CI)^a^
**Age**				
18–24	79(27.3)	39(13.5)	1.0	1.0
25–29	86(29.8)	99(34.3)	0.39(0.23–0.65)***	0.56 (0.29–1.06)*
30–34	77(26.6)	64(22.2)	0.58(0.34–0.99)*	0.82(0.41–1.64)
34–39	29(10.0)	48(16.6)	0.28(0.15–0.53)***	0.48(0.21–1.10)*
40+	18(6.2)	39(13.5)	0.22(0.11–0.45)***	0.33(0.14–0.79)**
**Marital status**				
Single	76(26.3)	49(17.0)	1.0	1.0
Married	191(66.1)	235(81.3)	0.43(0.29–0.64)**	0.51(0.30–0.88)*
**Education**				
None	59(20.4)	46(15.9)	1.0	1.0
Primary	114(39.5)	118(40.8)	0.73(0.45–1.19)	0.72(0.39–1.32
Secondary-S1-S4	98((33.9)	102(35.3)	0.72(0.44–1.19)	0.57(0.30–1.08)
A level& tertiary	18(6.2)	23(8.0)	0.60(0.29–1.24)	0.31(0.12–0.84)*
**Religion**				
Catholic	122(42.7)	95(32.9)	1.0	1.0
Protestant	80(28.0)	73(25.3)	0.82 (0.54–1.25)	1.00(0.59–1.70)
Muslim	54(18.9)	87(30.1)	0.47(0.30–0.74)**	0.52(0.29–0.91)*
Other	30(10.5)	34(11.8)	0.69(0.40–1.18)	0.86(0.45–1.64)
**Weekly Income in Shs (USD)**				
<30,000 (<13)	17(5.9)	71(24.7)	1.0	1.0
30,000–49,999(13–20)	48(16.6)	80(27.9)	2.42(1.22–4.83)*	2.26 (1.07–4.76)*
50,000–79,999(21–30)	119(41.2)	79(27.5)	5.97(3.12–11.4)***	5.55(2.74–11.24)***
80,000–99,999(31–40)	13(4.5)	17(5.9)	3.34(1.27–8.78)*	3.02(1.04–8.80)*
100,000+(41+)	92(31.8)	40(13.9)	12.93(5.95–28.08)***	12.91(5.62–29.65)***

^a^ Obtained after conditional logistic regression

* p<0.05

**p<0.01

*** p<0.001

**Fig 2:**
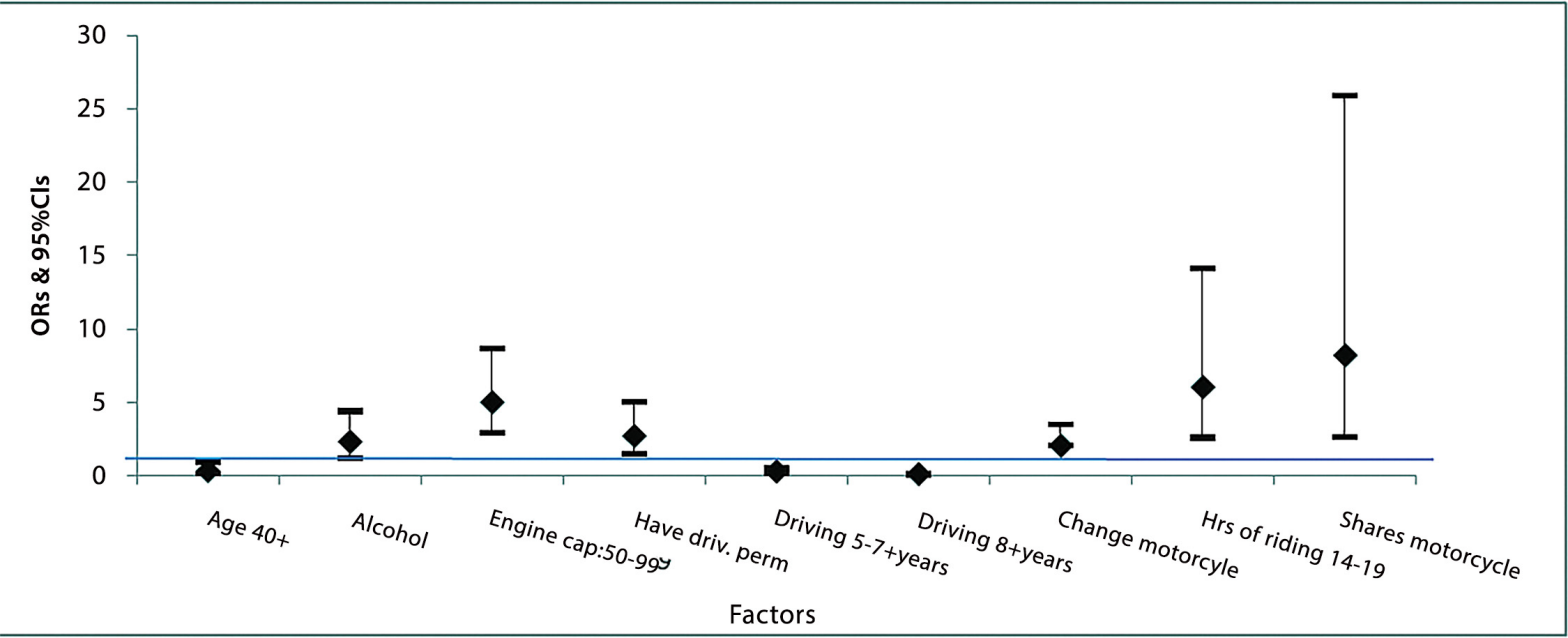
The third round of multivariable analysis included level of knowledge of traffic rules, Alcohol Use Disorders Identification Test score (AUDIT) and experience with police checks and riding till late (Table 5). Results from this analysis show that the odds of having an injury reduced with higher level of knowledge (OR = 2.05 for score 15–16 to OR = 0.09 for highest score-21-27). Police check for licenses, insurance, overloading was strongly associated with boda-boda injuries (OR = 3.19, 95%CI: 1.97–5.17). Odds for higher AUDIT score tended to rise with boda-boda injuries but the relationship slightly fell short of significance at 5% level. The odds of getting injured rose with the time of stopping riding (OR = 0.93 for those stopping at 8-9pm but rose to 3.39 for those stopping 10–12 midnight). The variables adjusted for but which did not significantly change the log likelihood were marital status, income, education and religion.

After controlling for all key variables, having an injury was strongly associated with younger age group, taking alcohol, lower engine capacity (<100cc), having a riding permit, having few years of riding experience (less than 3 years), not changing a motorcycle in past 1 year, riding for longer hours and sharing a motorcycle (Table 4 and Fig 2). Factors which were controlled for but never proved important in terms of change in log likelihood in last model were the marital status, income, education, religion, make of motorcycle (for example Bajaj make), number of motorcycles the rider owns, amount of money he makes, having other sources of income, frequency of riding, having taken a riding test, number of passengers, speed, wearing a reflective jacket, AUDIT score, score for knowledge of road safety rules and responsibilities, and encounter with traffic law enforcement.

**Table 4:** Multivariable analysis- all key variables.

Practices	Casesn (%)	Controln (%)	UOR^a^	AOR^a^
**Age**				
18–24	79(27.3)	39(13.5)	1.0	1
25–29	86(29.8)	99(34.3)	0.39(0.23–0.65)***	0.42(0.19–0.92)*
30–34	77(26.6)	64(22.2)	0.58(0.34–0.99)*	0.87(0.38–1.98)
34–39	29(10.0)	48(16.6)	0.28(0.15–0.53)***	0.39(0.14–1.10)
40+	18(6.2)	39(13.5)	0.22(0.11–0.45)***	0.30(0.09–1.00)*
**Alcohol consumption**				
No	203(70.2)	237(82.0)		1
Yes	86(29.8)	52(18.0)	1.97(1.31–2.96)**	2.30(1.19–4.45)*
**Engine Capacity**				
100+	122(42.2)	237(82.0)	1	1
50–99	167(57.8)	52(18.0)	5.11(3.41–7.66)***	5.03(2.91–8.70)***
**Have a driving permit**				
No	165(57.1)	180(62.3)	1	1
Yes	124(42.9)	109(37.2)	1.28(0.89–1.82)	2.71(1.44–5.07)**
**Number of years of riding**				
0–2	110(36.1)	37(12.8)	1.0	1
3–4	74(25.6)	62(21.5)	0.44 (0.26–0.76)**	0.29(0.12–0.67)**
5–7	73(25.3)	82(28.4)	0.31(0.18–0.54)***	0.25(0.11–0.56)***
8+	32(11.1)	108(37.4)	0.10(0.05–0.19)***	0.08(0.03–0.20)***
**Changed motorcycle in past 1 year**				
Yes	83(28.7)	126(43.6)	1	1
No	206(71.3)	163(56.4)	1.96(1.37–2.80)***	2.04(1.19–3.52)*
**Hours of riding**				
<12 hours	49(17.0)	67(23.2)	1	1
12 hours	42(14.5)	67(23.2)	0.96(0.55–1.68)	0.93 (0.41–2.10)
13 hours	51(17.7)	74(25.6)	0.95 (0.56–1.61)	1.28(0.63–2.61)
14–19 hours	147(50.9)	81(28.0)	2.53(1.55–4.11)***	6.05(2.58–14.18)**
**Shares motorcycle**				
No	246(85.1)	276(95.8)	1	
Yes	43(14.9)	12(4.2)	4.88(2.28–10.43)***	8.25 (2.62–25.9)***

^a^ Obtained after conditional logistic regression

*p<0.05

** p<0.01

***p<0.001

**Table 5:** Multivariable analysis- knowledge and other variables.

Practices	CasesN (%)	Control n (%)	UOR^a^	AOR^a^
**Age**				
18–24	79(27.3)	39(13.5)	1.0	1.0
25–29	86(29.8)	99(34.3)	0.39(0.23–0.65)***	0.37(0.18–0.80)*
30–34	77(26.6)	64(22.2)	0.58(0.34–0.99)*	0.54(0.25–1.16)
34–39	29(10.0)	48(16.6)	0.28(0.15–0.53)***	0.35(0.15–0.83)*
40+	18(6.2)	39(13.5)	0.22(0.11–0.45)***	0.30(0.12–0.80)*
**Level of knowledge of traffic rules & rider responsibility**				
10–14	68(23.8)	45(15.7)	1	1
15–16	86(30.1)	30(10.45)	2.38(1.17–4.83)*	2.05 (0.83–5.04)
17–18	74(25.9)	42(14.6)	1.07(0.60–1.90)	0.85(0.41–1.75)
19–20	39(13.6)	71(24.7)	0.42(0.23–0.77)**	0.57(0.27–1.20)
21–27	19(6.64)	99(34.49)	0.11(0.05–0.24)***	0.09 (0.04–0.21)***
**Ever been stopped by Police to check on insurance/ License /condition**				
No	81(28.0)	181(62.6)	1	1
Yes	208(72.0)	108(37.4)	3.56(2.50–5.08)***	3.19(1.97–5.17)***
**AUDIT Score**				
<8	247(85.5)	271(93.8)	1	1
8–15	27(9.34)	14(4.8)	1.93(1.01–3.68)*	1.48(0.57–3.83)
16+	15(5.2)	4(1.4)	3.75(1.24–11.30)*	4.65(0.96–22.55)
**End of riding**				
1:00–7:59pm	65(22.5)	101(35.0)	1.0	1.0
8:00–8:59pm	56(19.4)	88(30.5)	0.96(0.57–1.60)	0.93(0.44–1.96)
9:00–9:59 pm	46(15.9)	62(21.5)	1.09 (0.67–1.77)	1.02(0.52–2.01)
10–12 midnight	89(30.8)	32(11.1)	3.88(2.25–6.69)***	3.39(1.57–7.31)**
Morning next day	33(11.4)	6(2.1)	8.03(2.98–21.67)***	2.98(0.94–9.46)

^a^ Obtained after conditional logistic regression

*p<0.05

** p<0.01

***p<0.001

## Discussion

Results from the study have shown that injured motorcyclists were more likely to be younger, unmarried and earned a higher income compared to those who were not injured. Further on, the results show that factors independently associated with injury among the riders were alcohol consumption, lower engine capacity, having a driving permit, fewer years of riding experience, not changing a motorcycle in previous year, riding for a longer time in a day and sharing a motorcycle. Other factors that were significantly associated with boda-boda related injury but after adjusting for only baseline characteristics were low knowledge of road safety rules, being checked by police and having a higher alcohol use disorder test score

The difference in socio-demographic factors between the cases and controls in the study are consistent with results from several studies. For example, the protective effect of higher age was established in case control studies in New Zealand, Malaysia and Pakistan [5, 12, 32–33]. Not many studies have established the influence of marital status and income on road traffic injury among motorcycle riders but bivariate analysis in a study in Nigeria found that those who never married and those who had other jobs had higher odds of getting road traffic injuries compared to their counterparts[34]. The correlation of road injury with being unmarried can be explained by a tendency for young people to be more risk takers. They are more likely to disobey traffic rules[35], ride at a high speed, and take alcohol. Other studies have found a correlation between road injury among motorcyclists and lower education level which this study has not found very significant [12–14]. More research on influence of socio-demographic factors on injury among boda-boda riders in Uganda is needed since all but age were not significant in a multivariable model.

Results on association of road injuries among motorcycle riders with alcohol consumption and short riding experience concur with findings of studies carried out in several countries including the USA, New Zealand and France [13–14, 36–38]. Alcohol consumption impairs judgment and this explains the correlation with road injury. Consumption of alcohol can also work as a proxy for addictive behaviors such as smoking which have been found to be strongly correlated with motorcycle injuries[33]. Long riding experience is strongly associated with more discipline on the road, use of helmets and higher age. This is why it is not surprising that helmet use was ejected out of the multivariable model. It had a strong autocorrelation with age and experience of riding. Otherwise, despite its proven protective effect, helmet use in the country is low partly because of poor attitudes to its use and poor enforcement[39].

Higher odds of getting injured among those who have been stopped for routine checks by police may be explained by a possibility that those who are checked by police are more likely to be traffic offenders who are at high risk of getting injured. The issue of higher odds of injury among those with motorcycle riding permits needs further investigation because it’s unexpected. On cross tabulation with length of time of riding a motorcycle it was found that this unique relationship was most evident among those who had ridden motorcycles for less than 3 years. There has been a police campaign to test the riding competency of all commercial motorcyclists and this could have encouraged a rush for fake riding licenses.

Higher engine capacity has been associated with higher risk of injury among motorcyclists in a number of studies but this study has found different results. Examples of these studies are those by Langley et al in New Zealand [13], Cheng et al. and Krauss et al in the USA[17–18] and Adogu et al in Nigeria[40]. However, some studies have connected the higher engine capacity to severity of injuries rather than just a mere occurrence of injury[41–42]. This relationship between engine capacity and getting injured needs further research.

The association of road traffic injury with sharing motorcycles, not owning a motorcycle that one rides have also been established in several studies [5]. The association of sharing motorcycle with injury may be explained by lack of commitment to service the motorcycle, lack of experience of riding and lack of actual experience with that particular motorcycle. Sharing motorcycles may also be linked to situations when regular riders get tired at end of the day and friends or relatives take over the motorcycles and try to make extra income. These riders who take over are often learners, with less experience on the road. The protective effect of changing motorcycle in a year may be explained by strong association between low weekly income and changing motorcycles (p = 0.02). In the multivariable model of background characteristics higher weekly income was associated with higher odds of getting injured.

Association of working long hours, working till late and starting very early morning with injuries has featured in other studies in different countries [5, 13–14, 36–38]. The association can be explained partly by fatigue and poor visibility[43]. In this study, the number of riders that got injured was higher at around 6am, 12 noon and 8-10pm and clearly apart from 12 noon the rest of the times are dark. Slightly more than a half (54.3%) of the injuries occurred before 7am and after 7pm. The times between 7.00am and 9.00am, and 5.00pm and 8.00pm when the roads are heavily jammed do not seem to carry as much risk, possibly because the vehicles are barely moving, so any collisions that occur are low energy and do not result in injuries

Significant association of injury with lower level of knowledge of road safety rules, higher AUDIT score and having been stopped by traffic police officers can be explained by information on other factors discussed. Higher AUDIT score imply alcohol consumption which is already associated with higher odds of injury. Being stopped by police may signify a problem with riding motorcycle like influence of alcohol or risk taking which may cause an injury. The police may also stop a rider if he demonstrates ignorance of road safety rules which also can lead to injury.

### Generalisability of the results

The results of this study can be generalized to all major urban centers and other busy heavily populated areas in the country. Such places tend to have high concentration of commercial motorcyclists and are expected to have more riders injured on the road.

### Methodological Considerations

A rider who has not had an injury at around the same time his colleague is in hospital may not be the best control because he may have had an injury days or weeks before or after. In such circum stances the control is supposed to be among the cases. Selecting a control as a person who has not had an injury for a specified length of time may be better but such benefit could be eroded by inaccuracies.

## Conclusion and Recommendations

Major factors independently associated with road traffic injuries among boda-boda riders include young age, alcohol consumption, sharing motorcycles, working late hours, being young, lower engine capacity (below 100cc), short riding experience (less than 3 years), not changing a motorcycle in a year, and having a riding permit. Other factors found important include lower knowledge of road traffic rules and responsibilities, having higher score of alcohol use disorder score (AUDIT)

The research team makes a range of recommendations arising from the study results. Firstly there should be tougher controls for issuance of riding permits to young people aged less than 25 years. Secondly, current random drink-driving checks for motorists by traffic police should be extended to boda-boda riders. Investigators on this study are not aware of drink-riding checks carried out by the traffic police. Fourthly, consider screening for alcohol disorders among riders before they are hired or given permits.

Other recommendations call for more effort for road safety sensitization programs for boda-boda riders, investigation of validity of riding permits and more research. Sensitization programs should raise more awareness against sharing motorcycles, working long hours, working at times when visibility is low, and alcohol consumption. Information from the police force shows that there is an existing sensitization program for boda boda riders. The forces do also partner with the NGOs to carry out sensitization of the riders but they(riders) are too many[44]. The results of this study provide more areas of emphasis during these seminars and justify a need to have sensitization seminars held more regularly. They can reach more riders through their numerous associations. In connection to results that show association of rider experience of less than 3 years and having a road permit being liked to road injuries the police should investigate the validity of motorcycle riding licenses and test the riding competency of all who got licenses in 3 years before the study, that is around 2011–2014 periods. More research is needed to explain the risk of road traffic injury associated with sharing motorcycles, lower engine capacity and not changing motorcycles.

## Supporting Information

S1 Dataset Minimal data set with variables for the main final multivariable regression analysis. The data set is in STATA V12 format and has the variables case/control, age group, alcohol consumption, engine capacity, having a driving permit, length of experience of riding motorcycle, changing motorcycle in previous year, hours driven in a day, sharing a motorcycle and matched case identification number. The data set has been uploaded in the public repository Harvard dataverse and its URL is https://dataverse.harvard.edu/dataset.xhtml?persistentId=doi:10.7910/DVN/RSOQ5E.
(DTA)
